# Education influences the role of genetics in myopia

**DOI:** 10.1007/s10654-013-9856-1

**Published:** 2013-10-19

**Authors:** Virginie J. M. Verhoeven, Gabriëlle H. S. Buitendijk, Consortium for Refractive Error and Myopia (CREAM), Fernando Rivadeneira, André G. Uitterlinden, Johannes R. Vingerling, Albert Hofman, Caroline C. W. Klaver

**Affiliations:** 1 Department of Ophthalmology, Erasmus Medical Center, Room Na-2808, PO Box 2040, 3000 CA Rotterdam, The Netherlands; 2 Department of Epidemiology, Erasmus Medical Center, Rotterdam, The Netherlands; 3 Department of Internal Medicine, Erasmus Medical Center, Rotterdam, The Netherlands; 4 The Netherlands Consortium for Healthy Ageing, Netherlands Genomics Initiative, The Hague, The Netherlands

**Keywords:** Myopia, Refractive error, GxE, Gene-environment, Environmental factors

## Abstract

Myopia is a complex inherited ocular trait resulting from an interplay of genes and environmental factors, most of which are currently unknown. In two independent population-based cohorts consisting of 5,256 and 3,938 individuals from European descent, we tested for biological interaction between genetic predisposition and level of education on the risk of myopia. A genetic risk score was calculated based on 26 myopia-associated single nucleotide polymorphisms recently discovered by the Consortium for Refractive Error and Myopia. Educational level was obtained by questionnaire and categorized into primary, intermediate, and higher education. Refractive error was measured during a standardized ophthalmological examination. Biological interaction was assessed by calculation of the synergy index. Individuals at high genetic risk in combination with university-level education had a remarkably high risk of myopia (OR 51.3; 95 % CI 18.5–142.6), while those at high genetic risk with only primary schooling were at a much lower increased risk of myopia (OR 7.2, 95 % CI 3.1–17.0). The combined effect of genetic predisposition and education on the risk of myopia was far higher than the sum of these two effects (synergy index 4.2, 95 % CI 1.9–9.5). This epidemiological study provides evidence of a gene-environment interaction in which an individual’s genetic risk of myopia is significantly affected by his or her educational level.

**Electronic supplementary material:**

The online version of this article (doi:10.1007/s10654-013-9856-1) contains supplementary material, which is available to authorized users.

## Introduction

Myopia (nearsightedness) is the most common refractive error and one of the leading causes of blindness [1, 2]. Myopia currently affects more than one in four people in the United States and Western Europe [3], and has a prevalence higher than 70 % in urban areas in Asian countries [4, 5]. The global incidence of myopia is growing [6, 7], increasing the frequency of sight-threatening complications such as myopic macular degeneration, glaucoma, and retinal detachment [8–10].

Myopia is highly heritable; the risk of developing myopia is increased at least threefold among children with two myopic parents compared to children with no myopic parents [11, 12], and heritability estimates for refractive error range from 0.60 to 0.90 [13]. The Consortium for Refractive Error and Myopia (CREAM) and 23andMe independently conducted large genome-wide association studies, and identified more than 20 genetic loci for this trait [14–16]. Individuals with many risk variants at these loci have a tenfold increased risk of myopia [14].

Education is the most important environmental risk factor for myopia identified to date [17]. The risk of developing myopia is up to four times higher in persons with a university-level education compared to persons with only primary schooling [17]. Achieving a higher level of education requires many hours of intensive near work (up-close work)—particularly reading—and this may contribute to the increased relative risk of developing myopia. Indeed, an increase in the average population-wide educational level may have contributed to the recent rise in the prevalence of myopia [6, 7, 18]. There are hints that education may influence the effect of myopia genes, e.g., a study of an Amish population found that the refractive errors of well-educated carriers of the *MMP1* and *MMP10* risk variants tended to be more myopic than those of individuals with lower levels of education [19]. Whether this gene-education interaction plays a role in the entire spectrum of genetic variants is unknown.

We assessed the combined effect of genetic predisposition and educational level on the risk of myopia in two independent population-based cohorts from Rotterdam, the Netherlands. We computed a genetic risk score based on 26 established loci for refractive error, calculated mean refractive error as a function of genetic risk score for levels of education, estimated risk of myopia in combined strata of genetic risk and educational level, and examined biological interaction according to the synergy index developed by Rothman [20].

## Methods

### Study population

The study population consisted of participants from the Rotterdam Study cohorts who had baseline data on refractive error, educational level and genotype. All measurements were conducted after the Medical Ethics Committee of the Erasmus University had approved the study protocols and all participants had given a written informed consent in accordance with the Declaration of Helsinki. All participants were from European descent.

Rotterdam Study I (RS-I) was used as discovery cohort (Table 1). This prospective population-based cohort study included a total of 5,256 participants aged 55 years and older living in Ommoord, a suburb of Rotterdam, the Netherlands [21]. Baseline examinations took place between 1991 and 1993. Two independent Rotterdam Study cohorts were combined into a replication cohort (Table 1). The first cohort was Rotterdam Study II (RS-II), an independent cohort which included n = 1,984 participants aged 55+ years living in Ommoord since 2000 [21]. Baseline examinations took place between 2000 and 2002. The second cohort was Rotterdam Study III (RS-III), which included n = 1,954 participants aged 45+ years and older living in Ommoord since 2006 [21]. Baseline examinations took place between 2006 and 2009.

**Table 1:** Characteristics of the study population from all cohorts

	Discovery cohort	Replication cohort		Combined
	RS-I	RS-II	RS-III	RS-I, RS-II, RS-III
N	5,256	1,984	1,954	9,194
Sex (%), men (±SD)	42	46	44	43
Age, years (±SD)	68.4 ± 8.5	64.2 ± 7.5	59.1 ± 5.5	64.9 ± 9.2
Baseline examinations	1991–1993	2000–2002	2006–2009	1991–2009
Refractive error				
Mean refractive error, D (±SD)	0.85 ± 2.45	0.47 ± 2.51	−0.34 ± 2.61	0.52 ± 2.54
High myopia ≤−6D (%)	91 (1.7)	35 (1.8)	61 (3.1)	187 (2.0)
Medium myopia >−6D & ≤−3D (%)	268 (5.1)	145 (7.3)	240 (12.3)	653 (7.1)
Low myopia −3D & ≤−0.75D (%)	500 (9.5)	258 (13.0)	358 (18.3)	1,116 (12.1)
Emmetropia >−0.75D & <0.75D (%)	1,355 (25.8)	528 (26.6)	625 (32.0)	2,508 (27.3)
Low hyperopia ≥0.75D & <3D (%)	2,309 (43.9)	813 (41.0)	549 (28.1)	3,671 (39.9)
Medium hyperopia ≥3D & <6D (%)	661 (12.6)	187 (9.4)	104 (5.3)	952 (10.4)
High hyperopia ≥6D (%)	72 (1.4)	18 (0.9)	17 (0.9)	107 (1.2)
Educational level				
Primary education (%)	2,798 (53.2)	651 (32.8)	522 (26.7)	3,871 (43.2)
Intermediate education (%)	1,850 (35.2)	912 (46.0)	807 (41.3)	3,569 (38.8)
Higher education (%)	608 (11.6)	421 (21.2)	625 (32.0)	1,654 (18.0)
Genetic risk				
Mean genetic risk score (±SD)	2.7 ± 0.4	2.7 ± 0.4	2.7 ± 0.4	2.7 ± 0.34
Low genetic risk score (1.40–2.25) (%)	463 (8.8)	173 (8.7)	164 (8.4)	800 (8.7)
Mean N carried risk alleles (±SD)	17.7 ± 1.4	17.6 ± 1.4	17.6 (1.5)	17.7 ± 1.4
Medium genetic risk score (2.25–3.00) (%)	3,582 (68.2)	1,364 (68.8)	1,334 (68.3)	6,280 (68.3)
Mean N risk alleles (±SD)	22.7 ± 1.9	22.8 ± 2.0	22.7 (1.9)	22.8 ± 1.9
High genetic risk (3.00–4.00) (%)	1,211 (23.0)	447 (22.5)	456 (23.3)	2,114 (23.0)
Mean N risk alleles (±SD)	27.7 ± 1.7	27.7 ± 1.7	27.7 ± 1.7	27.7 ± 1.7

Values are mean ± standard deviation

*SD* standard deviation, *RS* Rotterdam study, *D* diopters

### Assessment of refractive error

All participants underwent a complete ophthalmological examination including a non-dilated measurement of refractive error of both eyes using a Topcon RM-A2000 auto refractor. Refractive error was analyzed as spherical equivalent, calculated according to the standard formula ‘SE = sphere + ½ cylinder’. Mean refractive error was calculated; when data from one eye was unavailable, the SE of the other eye was used. Exclusion criteria were (bilateral) cataract surgery and laser refractive procedures without knowledge of prior refraction, other refraction influencing intra-ocular procedures, keratoconus, and syndromes. Refractive error was categorized into high myopia [≤−6 diopters (D)], moderate myopia (>−6D & ≤−3D), low myopia (<−3D & ≤−0.75D), emmetropia (>−0.75D & <0.75D), low hyperopia (≥0.75D & <3D), medium hyperopia (≥3D & <6D), and high hyperopia (≥6D), using criteria defined by the CREAM consortium (CREAM consortium meeting, 2012, Sardinia, Italy).

### Assessment of educational level

Information on educational level was obtained during a home interview. Level of education was classified into: primary education (primary school or lower vocational education); intermediate education (lower secondary education or intermediate vocational education); and higher education (higher secondary education, vocational education, or university).

### Genotyping

We selected all 26 genome-wide significant single nucleotide polymorphisms (SNPs) associated with refractive error and myopia derived from a meta-analysis from the CREAM consortium involving a total of 45,758 study subjects [14]. SNP genotyping and imputation have been described in detail elsewhere [22]. Genotyping was performed using the Illumina Infinium II HumanHap550 chip v3.0 array (RS-I); the HumanHap550 Duo Arrays and the Illumina Human610-Quad Arrays (RS-II), and the Human 610 Quad Arrays Illumina (RS-III). For imputation, we used the Markov Chain Haplotyping (MACH) package version 1.0.15 software (imputed to plus strand of NCBI build 36, HapMap release #22, CEU panel). Most of the SNPs were genotyped or had a high imputation quality score (r^2^ ≥ 0.8).

### Genetic risk score

The genetic risk score was calculated based on all 26 SNPs using a previously reported weighting method [14]. Each SNP was weighted according to its relative effect size (β regression coefficient from CREAM meta-analysis, Supplementary Table 2). Genetic risk scores ranged from 1.4 to 4.0, with higher scores indicating a greater genetic predisposition to myopia. The genetic risk score was categorized into a low (1.4–2.25), medium (2.25–3.00) or high genetic load (3.00–4.00) based on the association with myopia (Supplementary Figure 1). We also calculated the number of risk alleles carried per individual (homozygote for the risk allele = 2 risk alleles, heterozygote = 1 risk allele, homozygote for the other allele = 0 risk alleles).

### Statistical analysis

Separate analyses were performed for the discovery cohort (RS-I), the replication cohort (RS-II and RS-III combined), and for the cohorts combined (RS-I, RS-II, and RS-III). First, we assessed independent associations between education and refractive error and myopia, and genetic risk score and refractive error and myopia using linear and logistic regression. Second, we examined the continuous relation between genetic risk score, level of education and refractive error by calculating mean refractive error and the regression coefficients β per genetic risk score category, stratified by level of education, and tested for significant differences between groups with a one way ANOVA F test. Third, we assessed the risk of moderate to high myopia (refractive error ≤−3.0 D) versus moderate to high hyperopia (refractive error ≥3.0 D) for combined strata of genetic risk score and educational level with logistic regression analyses, using low genetic risk score and primary education as the reference, adjusting for age and sex. These analyses were also performed using moderate to high myopia (refractive error ≤−3.0 D) versus emmetropia (refractive error >−0.75D & <0.75D) as the outcome.

We tested for biological interaction between genetic predisposition and education by calculating the age and sex adjusted synergy index (SI) according to Rothman [20]. This measures deviation from additivity of 2 factors, and is based on the ratio of the combined effect to the sum of the separate effects. A synergy index of more than 1.0 suggests that the effect of both factors together is greater than the sum of the effect of the separate factors.

All reported *P* values are nominal and two-sided. We used SPSS version 20.0.0 (SPSS Inc.) for all analyses.

## Results

Demographics of the study participants in the discovery (RS-I) and in the replication (RS-II and RS-III combined) cohorts can be found in Table 1. In all cohorts, the majority of subjects were low hyperopic or emmetropic; the mean refractive error was 0.52 D (SD 2.54). Primary or intermediate educational level was most common, although its relative proportion was highest in the discovery cohort (RS-I) (Table 1). The genetic risk score ranged from 1.4 to 4.0 with a mean of 2.7 (SD 0.4), corresponding to a range of 12–35 carried risk alleles, and a mean of 23.4 (SD 3.3) risk alleles per subject. The genetic risk score had identical distributions across all cohorts (Table 1). Both educational level and the genetic risk score were significantly associated with refractive error and myopia (*P* < 0.0001, Table 2).

**Table 2:** Association with refractive error and risk of myopia for genetic risk score and level of education

	Refractive error				Myopia			
	n	β	se	*P* value	n	OR	95 % CI	*P* value
Education								
Discovery cohort (RS-I)	5,256	−0.48	0.05	<0.0001	1,092	2.3	1.9–2.8	<0.0001
Replication cohort (RS-II & RS-III)	3,938	−0.58	0.06	<0.0001	807	2.2	1.7–2.7	<0.0001
Combined (RS-I, RS-II, RS-III)	9,194	−0.55	0.04	<0.0001	1,899	2.3	12.0–2.6	<0.0001
Genetic risk score								
Discovery cohort (RS-I)	5,256	−0.67	0.06	<0.0001	1,092	2.4	1.9–3.1	<0.0001
Replication cohort (RS-II & RS-III)	3,938	−0.72	0.07	<0.0001	807	3.1	2.3–4.2	<0.0001
Combined (RS-I, RS-II, RS-III)	9,194	−0.69	0.05	<0.0001	1,899	2.7	2.2–3.2	<0.0001

Beta regression coefficients of the association with refractive error were calculated using linear regression analyses. The risk of myopia (defined as refractive error ≤−3 diopters) were calculated using logistic regression analyses with hyperopia (defined as a refractive error ≥3 diopters) as a reference. Analyses for education were corrected for age, sex, and genetic risk score. Analyses for the genetic risk score were corrected for age, sex, and education

*β* beta regression coefficient in diopter, *se* beta standard error, *OR* odds ratio, 95 % *CI* 95 % confidence interval, *RS* Rotterdam study

**Fig. 1:**
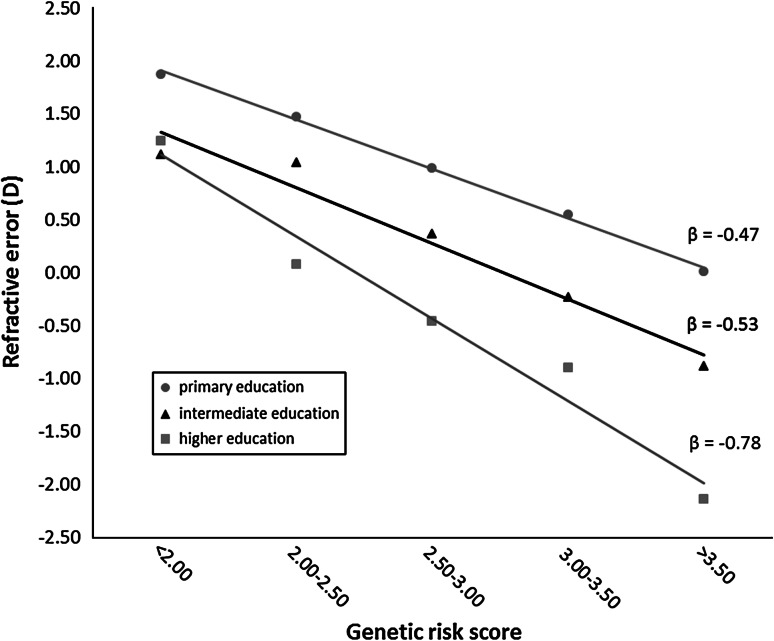
Refractive error as a function of genetic risk score stratified by level of education. Mean refractive error was calculated for each genetic risk score category and presented according to educational level. Regression lines were plotted, and the regression coefficient (β) is indicated for each line. The data are shown for the combined cohort (including RS-I, RS-II, and RS-III). The differences between educational level groups were statistically significant (*P* < 0.0001) for the discovery, replication and combined cohorts

The continuous relation between genetic risk score and refractive error stratified by level of education for the combined cohorts is shown in Fig. 1. Subjects who received a university or higher vocational education had a lower mean refractive error with increasing genetic risk than subjects with intermediate-level or primary education. These differences were statistically significant (β_high education_ = −0.78; β_intermediate_ = −0.53; β_primary_ = −0.47; *P* < 0.0001 for both the discovery and replication cohorts). Among individuals with the highest genetic risk, the refractive error averaged −2 diopters for high educational level, −0.8 diopters for intermediate education, and 0 diopters or emmetropia for primary schooling.

**Fig. 2:**
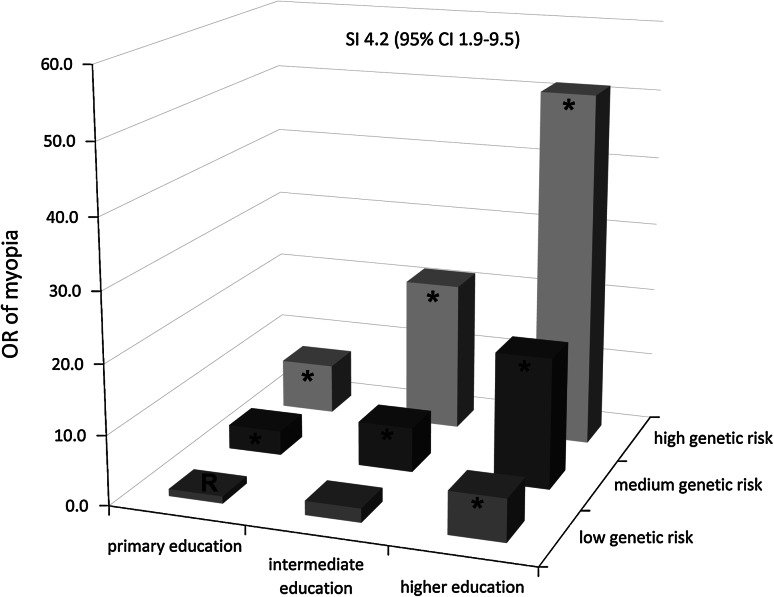
Risk of myopia for educational level and genetic risk score. The age- and sex-adjusted odds ratio for myopia (defined as a refractive error ≤−3 diopters) versus hyperopia (defined as a refractive error ≥3 diopters) for educational level and genetic risk score are plotted for the combined cohort (including RS-I, RS-II, and RS-III). The group with low genetic risk and primary education served as the reference. *, significant OR compared to the reference group; *SI* synergy index, *95* % *CI* 95 % confidence interval, *OR* odds ratio, *R* reference (i.e., OR 1.0)

We then estimated the risk of myopia for the combined strata of genetic risk and educational level (Table 3; Fig. 2). In both the discovery and replication cohorts, the risk of myopia among subjects with a high genetic risk score and high educational level was highly increased (OR_combined_ = 51.3; 95 % CI 18.5–142.6), and far higher than the sum of the risks among individuals with only one of these two factors (OR_combined_ for primary education = 6.1, 95 % CI 2.1–17.6.; OR_combined_ for high genetic risk = 7.2, 95 % CI 3.1–17.0).

**Table 3:** Risk of myopia for educational level and genetic risk score, adjusted for age and sex

	Primary education			Intermediate education			Higher education			*P* value for trend
	n	OR	95 % CI	n	OR	95 % CI	n	OR	95 % CI	
Discovery cohort, RS-I (n = 1,092)										
Low genetic risk	65	1.0 (reference)	na	42	4.3	1.1–17.1	14	5.9	1.1–30.9	0.001
Medium genetic risk	386	4.6	1.4–15.1	268	9.1	2.7–29.9	88	23.5	6.7–82.2	<0.0001
High genetic risk	105	8.4	2.4–28.9	93	26.5	7.6–91.5	31	71.6	15.6–328.3	<0.0001
SI 5.5; 95 % CI 1.6–18.5										
Replication cohort, RS-II & RS-III (n = 807)										
Low genetic risk	23	1.0 (reference)	na	24	0.7	0.1–3.8	20	5.5	1.3–23.4	0.04
Medium genetic risk	140	2.8	0.8–8.9	233	4.6	1.5–14.3	164	14.6	4.5–47.3	<0.0001
High genetic risk	50	7.5	2.1–26.1	92	19.0	5.6–64.8	61	37.2	9.1–152.3	<0.0001
SI 3.3; 95 % CI 1.1–9.9										
Combined cohorts, RS-I, RS-II, RS-III (n = 1,899)										
Low genetic risk	88	1.0 (reference)	na	66	2.0	0.7–5.5	34	6.1	2.1–17.6	0.008
Medium genetic risk	526	3.5	1.5–7.9	501	6.4	2.9–14.4	252	18.8	8.1–43.7	<0.0001
High genetic risk	155	7.2	3.1–17.0	185	21.6	9.2–50.6	92	51.3	18.5–142.6	0.007
SI 4.2; 95 % CI 1.9–9.5										

Myopia was defined as a refractive error ≤−3 diopters. For this analysis, subjects with hyperopia (defined as refractive error ≥3 diopters) were used as controls

*OR* odds ratio, 95 % *CI* 95 % confidence interval, *SI* synergy index, *RS* Rotterdam study

The synergy index according to Rothman [20] was statistically significant in both the discovery cohort and the replication cohort (SI_combined_ = 4.2; 95 % CI 1.9–9.5), indicating a biological interaction (Table 3).

The risks in the combined strata using myopia versus emmetropia as the outcome showed similar trends, however, ORs were lower in all strata and the synergy index did not reach statistical significance (Supplementary Table 1, Supplementary Figure 2).

## Discussion

In two independent cohorts from the population-based Rotterdam Study, we found a significant biological interaction between education and genetic risk of myopia as represented by 26 associated SNPs [14]. Subjects with high genetic risk in combination with high levels of education had a far higher risk of myopia than subjects with only one of these two factors. We observed this effect in both quantitative analyses with refractive error in diopters as a continuous outcome, as well as in qualitative analyses comparing the extreme ends of the physiological spectrum. The interaction effect of genetic predisposition and education on myopia risk was more than 4 times higher than the sum of the separate effects.

Our study has specific strengths. First, the size of the combined study population and the frequency of exposures and outcomes were sufficiently high to detect a biological interaction. In addition, the interaction and the risk estimates were significant in the discovery cohort and were confirmed in the replication cohort, suggesting high reliability of these results. On the other hand, our study was limited by the rough approximations of the two risk factors (genetic risk and education level). Our genetic risk score was based on 26 myopia risk SNPs which were identified by the CREAM consortium, and of which 14 were also found by 23andMe (15). The effect sizes of the remaining 8 23andMe top hits were very small (betas between 0.03 and 0.08), and incorporation of these SNPs did not change our findings. Nevertheless, more in-depth knowledge regarding the genetic background of myopia in the future will improve precision of the effect sizes. In addition, education may be an even stronger effect modifier when absolute years of education can be incorporated. Finally, we observed a cohort effect that merits mention. Subjects from the RS-I study (which covered the period 1991 through 1993 and included subjects age 55 years and older) generally had a lower educational level than subjects from the RS-III study (which covered the period from 2006 through 2009 and included subjects age 45+ years). However, because the interaction effect of education and genetic risk was detected independently in each of these cohorts, this cohort effect did not likely affect our findings.

What mechanisms might underlie this strong interaction between education and genetic risk? Achieving higher levels of education requires more intensive near work. Several studies have reported that near work is directly related to the development of myopia by causing retinal defocus and degradation of retinal image contrast, which can subsequently trigger eye growth as a compensatory mechanism [23–27]. However, others point out that persons with a higher educational level are at risk of myopia because they spend less time outside [28]. Education may reflect a complex combination of these factors, ultimately leading to up-regulation of risk genes, excessive eye growth and development of myopia.

The 26 recently discovered SNPs are present in genes involved in various processes, including neurotransmission, ion channel function, extracellular matrix formation and stabilization, retinoic acid metabolism, and ocular development. As with gene-environment interactions described for other disorders [29], it is unlikely that all of these genes contribute to the gene-education interaction in myopia. We hypothesize that neurotransmission-related genes that are expressed in the outer retina may be particularly vulnerable to the effect of retinal defocus, in contrast to developmental eye genes and genes involved in the extracellular matrix. A genome-wide analysis of SNP-education interaction in a large study population might reveal the modifying effects of individual SNPs.

Interestingly, a combined effect between near work and outdoor activity—a known protective factor against myopia—has also been reported [28]. In addition, several studies have reported that outdoor activity can counteract the increased risk from near work [28, 30, 31]. Whether this type of work can also reduce the risk of near work among individuals at high genetic risk is an interesting question that merits investigation.

Genetic research regarding myopia has traditionally been guided by the assumption that genes exert a direct effect on the trait. Our finding of a robust gene-environment interaction casts new light on the current evolutionary model and offers new opportunities to identify additional myopia genes. Working many hours at near work tasks appears to be the requisite trigger for eliciting strong gene effects, and once this condition is satisfied, the genes become highly penetrant. We recommend that the search for new myopia genes should focus on study participants who are selected based on exposure (i.e., subjects with a high level of education and/or intensive near work work). This approach can also be readily extended to the study of other complex disorders. If environmental exposures show considerable variation within the study sample, genes might account for only a small percentage of the phenotypic variation. However, if these exposures have low variability among the study cohort, a disease that had previously been believed to arise from many small genetic effects might actually be caused by only a few genes, each of which exerts a relatively large effect.

Traditionally, analyzing gene-environment interactions has been extremely challenging, and this is primarily because the low relative frequencies of the exposures and/or trait have limited the study’s statistical power [32]. However, given that our analysis has overcome these limitations, this approach may serve as a textbook example of biological interactions between genes and the environment.

## Conclusion

This epidemiological study provides evidence of gene-by-environment (GxE) interaction, in which an individual’s genetic risk of myopia is affected by his or her educational level. Subjects with many variants in myopia genes and a higher educational level (e.g. university) are much more susceptible to develop myopia than those with only one of these two factors. Education may reflect a complex combination of higher level of reading exposure and corresponding lower levels of outdoor physical activity, ultimately leading to up-regulation of risk genes, excessive eye growth and the development of myopia.

## Electronic supplementary material

Below is the link to the electronic supplementary material.

Supplementary material 1 (DOCX 380 kb)
