# Supplementary material for: Education influences the role of genetics in myopia
Source: Eur J Epidemiol. 2013 Oct 19;28(12):973–80. doi: 10.1007/s10654-013-9856-1 (PMC3898347; doi:10.1007/s10654-013-9856-1)
Supplement: Supplementary file 1 — Supplementary material 1 (DOCX 380 kb) [file 10654_2013_9856_MOESM1_ESM.docx]

**Supplementary material**

**Education influences the role of genetics in myopia**

**Authors**

Virginie J. M. Verhoeven^1,2^, Gabriëlle H. S. Buitendijk^1,2^, Consortium for Refractive Error and Myopia (CREAM)^#^, Fernando Rivadeneira^2,3,4^, André G. Uitterlinden^2,3,4^, Johannes R. Vingerling^1,2^, Albert Hofman^2,4^, Caroline C. W. Klaver^1,2,^*

**Affiliations**

1. Department of Ophthalmology, Erasmus Medical Center, Rotterdam, The Netherlands
2. Department of Epidemiology, Erasmus Medical Center, Rotterdam, The Netherlands
3. Department of Internal Medicine, Erasmus Medical Center, Rotterdam, The Netherlands
4. The Netherlands Consortium for Healthy Ageing, Netherlands Genomics Initiative, The Hague, The Netherlands

^#^A full consortium membership list appears in the Supplementary material.

*Corresponding author:

Erasmus Medical Center, room Na-2808, PO Box 2040, 3000 CA, Rotterdam, the Netherlands, e-mail: [c.c.w.klaver@erasmusmc.nl](mailto:c.c.w.klaver@erasmusmc.nl), phone number +31651934491, fax number +31107044657

**Conflict of interest statement**

The authors declare no conflict of interest.

**Supplementary Table 1. Risk of myopia for educational level and genetic risk score, adjusted for age and sex**

Myopia was defined as a refractive error ≤-3 diopters. For this analysis, subjects with emmetropia (defined as refractive error >-0.75 D & SE <0.75 diopters) were used as controls.

The risks in the combined strata using myopia versus emmetropia as the outcome showed similar trends as myopia versus hyperopia analyses, however, ORs were lower in all strata and the synergy index did not reach statistical significance.

OR, odds ratio; 95% CI, 95% confidence interval; SI, synergy index; RS, Rotterdam Study.

**Supplementary Table 2. Summary of the SNPs used for calculation of the genetic risk score**

Summary of the 26 myopia SNPs that were used to calculate the genetic risk score. OR was calculated for myopia (SE ≤ -3 D) versus hyperopia (SE ≥ 3 D). All SNPs had the same minor allele in all cohorts. SNP, single nucleotide polymorphism; nearest gene, reference NCBI build 37; A1, risk allele; A2, other allele, MA, minor allele; MAF, average minor allele frequency; beta, effect size on refractive error in diopters based on allele A1; OR, odds ratio for myopia versus hyperopia; RS, Rotterdam Study.

**Supplementary Figure 1. Genetic risk score for myopia**


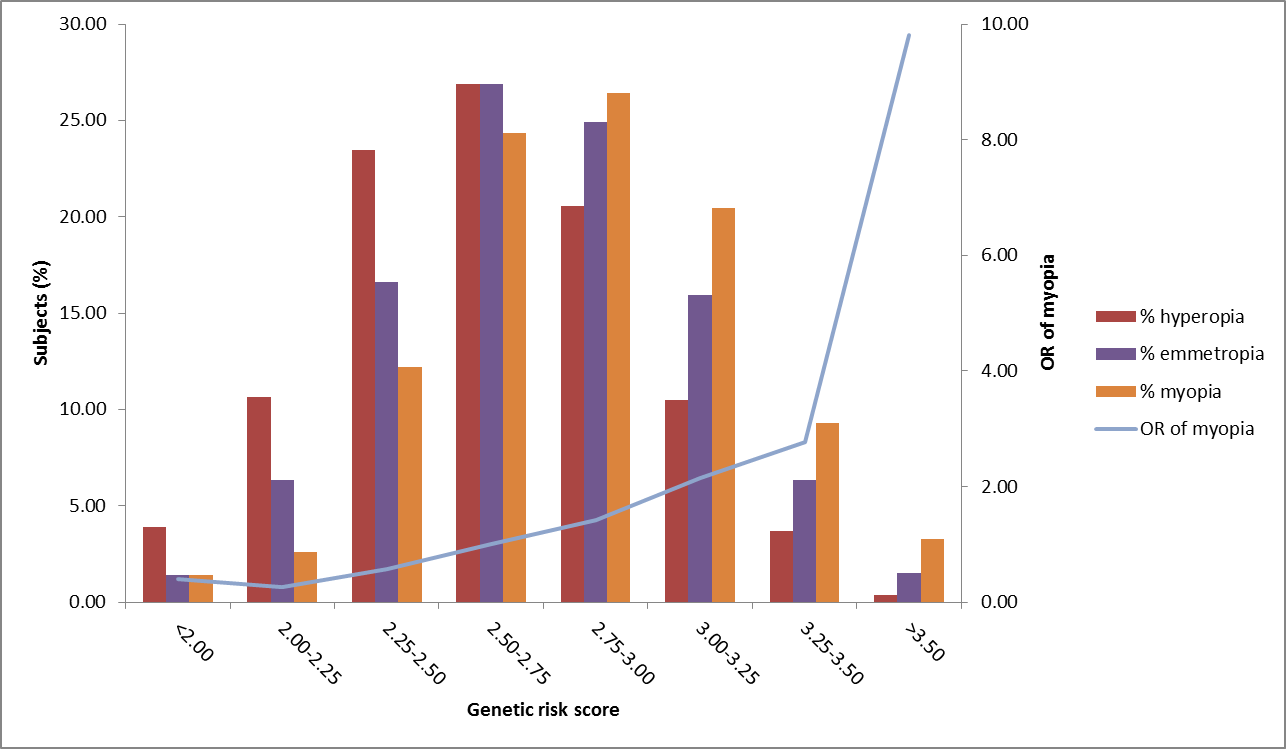


This figure was taken from Nature Genetics.[[1](#_ENREF_1)] Distribution of subjects from RS-I, RS-II and RS-III combined (n = 9,307) with myopia (SE ≤ −3 D, emmetropia (SE ≥ −1.5 D and ≤ 1.5 D) and hyperopia (SE ≥ 3 D) as a function of the genetic risk score. The genetic risk score was calculated based on all 26 SNPs using a previously reported weighting method.[[1](#_ENREF_1)] Each SNP was weighted according to its relative effect size (β coefficient from CREAM meta-analysis, **Table S2**). Based on the odds ratio (OR) in this figure, the genetic risk score was categorized into a low (1.4-2.25), medium (2.25-3.00) or high genetic load (3.00-4.00).

**Supplementary Figure 2. Risk of myopia for educational level and genetic risk score**


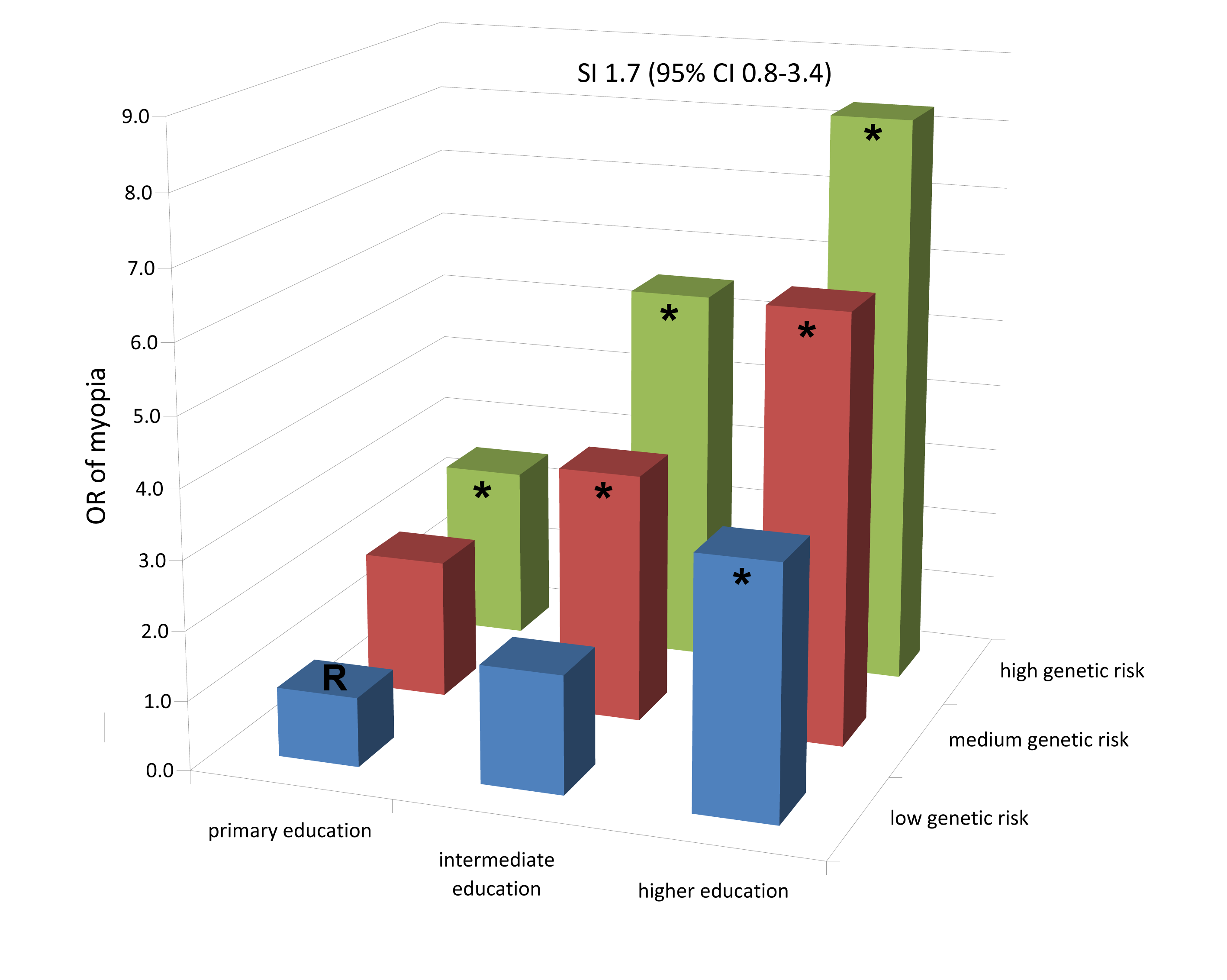


The age- and sex-adjusted odds ratio for myopia (defined as a refractive error ≤-3 diopters) versus emmetropia (defined as a refractive error >-0.75 D & <0.75 diopters) for educational level and genetic risk score are plotted for the combined cohort (including RS-I, RS-II, and RS-III). The group with low genetic risk and primary education served as the reference.

*, significant OR compared to the reference group; SI, synergy index; 95% CI, 95% confidence interval; OR, odds ratio; R, reference (i.e., OR = 1.0).

**Consortium for Refractive Error and Myopia (CREAM) membership list**

1958 British Birth Cohort - Jugnoo S. Rahi, Pirro G. Hysi
Aichi cohort - Nagahisa Yoshimura, Kenji Yamashiro, Masahiro Miyake
ALIENOR - Cécile Delcourt, Cecilia Maubaret
ALSPAC - Cathy Williams, Jeremy A. Guggenheim

ANZRAG - Jamie E. Craig, Kathryn P. Burdon, Rhys D. Fogarty
AREDS1a - Sudha K. Iyengar, Robert P. Igo Jr, Emily Chew, Sarayut Janmahasathian
AREDS1b - Sudha K. Iyengar, Robert P. Igo Jr, Emily Chew, Sarayut Janmahasathian
AREDS1c - Dwight Stambolian, Joan E. Bailey Wilson
BATS - Nick Martin, Yi Lu
Beijing Eye Study - Ya Xing Wang, Liang Xu, Seang-Mei Saw
Blue Mountain Eye Study - Paul N. Baird, Maria Schache, Paul Mitchell, Jie Jin Wang
CIEMS - Jost B. Jonas, Vinay Nangia
CROATIA-Korčula - Caroline Hayward, Alan F. Wright, Veronique Vitart
CROATIA-Split - Ozren Polasek, Harry Campbell, Veronique Vitart
CROATIA-Vis - Igor Rudan, Zoran Vatavuk, Veronique Vitart
DCCT - Andrew D. Paterson, S. Mohsen Hosseini
Duke FECD Fuchs Dystrophy GWAS - Sudha K. Iyengar, Robert P. Igo Jr, Jeremy R. Fondran
Duke Myopia Study - Terri L. Young, Sheng Feng
Erasmus Rucphen Family Study - Virginie J.M. Verhoeven, Caroline C. Klaver, Cornelia M. van Duijn
Estonian Genome Project / EGCUT - Andres Metspalu, Toomas Haller, Evelin Mihailov
FITSA - Olavi Pärssinen, Juho Wedenoja
Framingham Eye Study - Joan E. Bailey Wilson, Robert Wojciechowski
GEMT - Paul N. Baird, Maria Schache
Gutenberg Health Study - Norbert Pfeiffer, René Höhn
Hong Kong cohort study - Chi Pui Pang, Li Jia Chen, Pancy O. Tam, Vishal Jhanji, Alvin L. Young
KORA - Thomas Meitinger, Konrad Oexle, Aharon Wegner
Kyoto high myopia - Nagahisa Yoshimura, Kenji Yamashiro, Masahiro Miyake
LIKI - Olavi Pärssinen
Myopia Genomics Study (Hong Kong HTI) - Shea Ping Yip, Daniel W. H. Ho
Ogliastra Genetic Park Study - Mario Pirastu, Federico Murgia, Laura Portas, Genevra Biino
ORCADES - James F. Wilson, Brian Fleck, Veronique Vitart
Penn Family Studies - Dwight Stambolian, Joan E. Bailey Wilson
RAINE - Alex W. Hewitt, Wei Ang
Rotterdam Study - Virginie J.M. Verhoeven, Caroline C. Klaver, Cornelia M. van Duijn
SCES - Seang-Mei Saw, Tien-Yin Wong, Yik-Ying Teo, Qiao Fan, Ching-Yu Cheng, Xin Zhou, M. Kamran Ikram
SCORM - Seang-Mei Saw, Yik-Ying Teo, Qiao Fan, Ching-Yu Cheng, Xin Zhou, M. Kamran Ikram
SIMES - Seang-Mei Saw, Tien-Yin Wong, Yik-Ying Teo, Qiao Fan, Ching-Yu Cheng, Xin Zhou, M. Kamran Ikram
SINDI - Seang-Mei Saw, Tien-Yin Wong, Yik-Ying Teo, Qiao Fan, Ching-Yu Cheng, Xin Zhou, M. Kamran Ikram
SP2 - Seang-Mei Saw, E-Shyong Tai, Yik-Ying Teo, Qiao Fan, Ching-Yu Cheng, Xin Zhou, M. Kamran Ikram
STARS - Seang-Mei Saw, Yik-Ying Teo, Qiao Fan, Ching-Yu Cheng, Xin Zhou, M. Kamran Ikram
TEST - David A. Mackey, Stuart MacGregor
TwinsUK - Christopher J. Hammond, Pirro G. Hysi
Utah Timorese - Margaret M. Deangelis, Margaux Morrison
Wenzhou - Xiangtian Zhou, Wei Chen
WESDR - Andrew D. Paterson, S. Mohsen Hosseini
Yokohama Study - Nobuhisa Mizuki, Akira Meguro
Young Finns Study - Terho Lehtimäki, Kari-Matti Mäkelä, Olli Raitakari, Mika Kähönen

**References**

1. Verhoeven VJ, Hysi PG, Wojciechowski R, Fan Q, Guggenheim JA, Hohn R et al. Genome-wide meta-analyses of multiancestry cohorts identify multiple new susceptibility loci for refractive error and myopia. Nat Genet. 2013;45(3):314-8. doi:ng.2554 [pii]

10.1038/ng.2554.
